# Unsuspected Diagnosis of Uterine Leiomyosarcoma after Laparoscopic Myomectomy in an Isolated Bag

**DOI:** 10.1155/2018/6342081

**Published:** 2018-06-14

**Authors:** Süleyman Salman, Fatma Ketenci Gencer, Bülent Babaoğlu, Melih Bestel, Serkan Kumbasar, Guray Tuna, Esra Güzel, Durkadın Elif Yıldız, Tuba Kotancı, Ali Selçuk Yeniocak, Özlem Sögüt

**Affiliations:** Gaziosmanpaşa Taksim Eğitim ve Araştırma Hastahanesi, Kadın Hastalıkları ve Doğum Bölümü, İstanbul, Turkey

## Abstract

Minimally invasive techniques are generally applied for patients suspected of having benign fibroids if medical treatment is insufficient. On the other hand, sometimes some occult carcinomas of uterus like leiomyosarcomas may be reported for the patients' applied morcellation. This condition is rare but outcomes are clinically significant. Fragmentation of occult sarcoma in the abdominal cavity without isolation bag results in widespread and poor survival. In this article, we report a case of 37-year-old woman suffering from pain due to unexpected leiomyosarcoma. Laparoscopic myomectomy was performed with power morcellation in an isolated bag. Although isolation bag is generally reported to be preventive, recurrence of sarcoma was seen at 5th month of follow-up. Even though morcellation within a bag seems to block wide spreading, dispersion of tumor cannot be stopped and more investigations have to be done.

## 1. Introductıon

Uterine sarcomas comprise 3% of uterine malignancies and leiomyosarcoma (LMS) accounts one-third of all uterine sarcomas [1]. LMS is a significant reason for uterine cancer deaths and its five-year overall survival rates range from 30% to 42% [1, 2]. The patient age is the major risk factor and patients older than 55 are at increased risk [1]. On the other hand LMS cannot be diagnosed accurately by ultrasound, computed tomography (CT), and magnetic resonance imaging (MRI) without diffuse weighted imaging [3]. Patients may undergo surgical procedure due to supposed benign fibroids which are in fact malignant. Minimally invasive techniques such as laparoscopic myomectomy or hysterectomy with morcellation for supposed benign leiomyosarcomas are nowadays controversial according to US Food and Drug Administration Safety communication initially issued in April 2014 and updated in November 2014[4].

**Figure 1:**
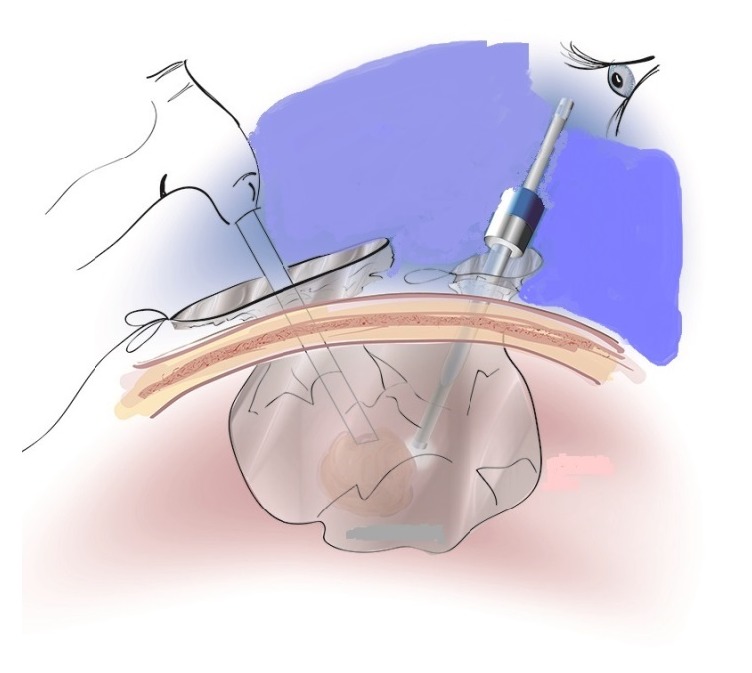
Isolated bag.

We report a case of 37-year-old young women with presumed benign leiomyoma treated with laparoscopic myomectomy with morcellation within an isolated morcellation bag reported as leiomyosarcoma at paraffin section (Figure 1).

## 2. Case Report

37-year-old woman presented to the emergency room with headache. Bilateral pelvic sensitivity and a mass painful to touch in a plastic texture that filled the Douglas' pouch were detected during the examination; the mass was evaluated in favor of leiomyoma. ß-hcg was negative, wbc was 9800/mm^3^, hgb was 12 g/dl, htc was 35%, plt was 282000/mm^3^, and no unusual characteristics was found in complete urinalysis. Ultrasonography revealed a mass consistent with a degenerated myoma measuring 77x82mm, of which subserous component was greater in the posterior wall and endometrial thickness was 7-8 mm, which was concordant with the cycle. The left ovary of the patient was normal, but since the right ovary could not be fully evaluated, computerized tomography (CT) was requested for possible adnexal pathologies. CT demonstrated a hypodense nodular lesion with a diameter of 75 mm that extended to the right adnexal region and MRI with contrast was performed on the patient. MRI revealed a mass with a hypovascular appearance following a heterogeneous and hypointense IV contrast material with a diameter of 8 mm, which appeared to displace the posterior cervix.

**Figure 2:**
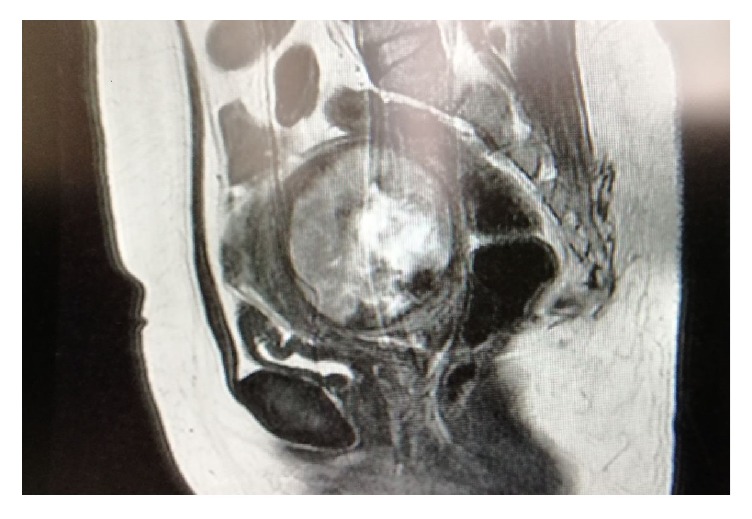
MRI image of myoma before surgery.

It is seen that myoma included by central necrosis depleted by cervix in the posterior part of the uterus. Also the development of necrosis was seen and myomatosis was evaluated in favor of degeneration (Figure 2).

**Figure 3:**
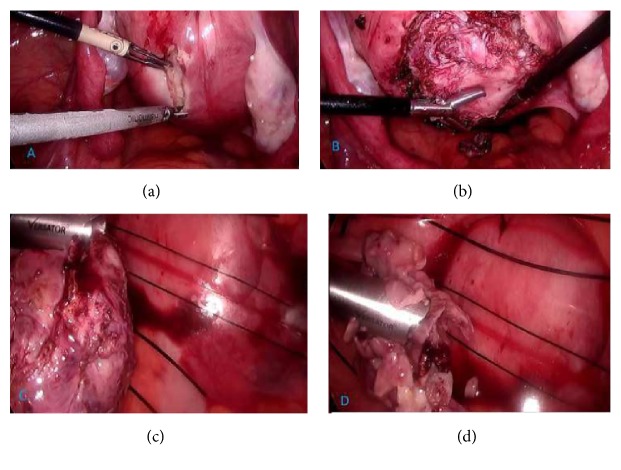
(a, b, c, d)   Laparoscopic morcellation of fibroid in isolated bag.

In addition, since the patient's pain regressed spontaneously during the follow-up, surgical operation was postponed to perform in elective conditions. After making necessary preparations for the operation, laparoscopic myomectomy was performed. Myoma was not considered suspicious apart from being degenerated. It was removed by morcellating in an isolated bag (Figures 3(a), 3(b), 3(c), and 3(d)).

No complication or hemorrhage occurred during surgery. As there was not any problem observed during the post-op follow-up, the patient was discharged from the hospital on condition that the pathology report was brought on the second day of post-op. The subsequent report revealed marked cellularity and pleomorphism, extensive necrosis, mitosis >5/10BBA, KI67 proliferation index >%50, weak positivity for p53, and LMS with no detectable lymphovascular invasion.

The patient was informed about the pathology report and interned again to perform transabdominal hysterectomy and bilateral salphingoopherectomy. The pathology report revealed “atypical pleomorphic fusiform cell proliferation area” measuring 3x2 mm in a focal area within the cavity consistent with residual LMS when evaluated together with laparoscopic myomectomy material.

**Figure 4:**
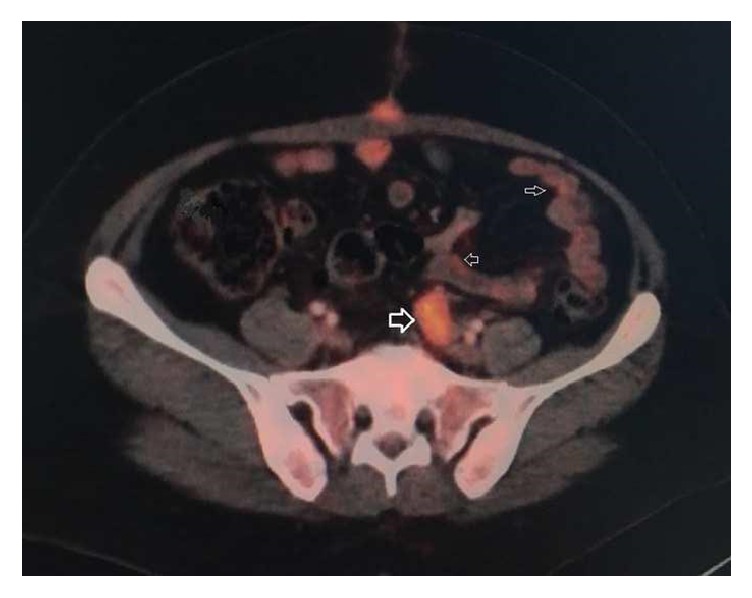
Abdominal positron emission tomographic (PET-CT) scan. Axial view shows variable-sized heterogeneous contrast-enhancing above rectosigmoid mesentery towards the left abdominal wall in pelvic floor (arrowheads). Arrow shows the lateral port site deposit.

To follow-up the patient was referred to radiotherapy and medical oncologists. After 3 months from her 1^st^ surgery, positron emission tomographic scan (PET-CT) was performed and fluoro-2-deoxyglucose (FDG) uptake is noted in the area above rectosigmoid mesentery towards the left abdominal wall in pelvic floor (Figure 4).

She was referred to gynecologic oncologist and 3^rd^ operation was performed. A mass about 4-5 cm of size in defined localization was excised; there were not any other mass and lymphadenopathy detected during the surgery. The pathology result was high grade malign mesenchymal tumor with high cellularity, extensive necrosis, mitosis 40/10 BBA, and moderate atypia. There was no tumor in surgical margin. She is now being followed up by the gynecologic oncologist with appropriate medical treatment.

## 3. Discussion

Myomectomy is a surgical approach for uterine fibroids for young patients unresponsive to medical treatment [4]. Uterine fibroids, in other words leiomyomas, affect not only women's health but also quality of life and fertility [5]. Minimally invasive procedures with morcellation which means fragmentation into small pieces make taking out large leiomyomas or uterine specimens easier [6]. LMS is reported after surgery for supposed benign leiomyoma in 1 case per 1960-8300 fibroid surgeries [7]. Risk of occult malignancy is 20 times higher between 50 and 59 aged women when compared to those younger than 40 [8]. In our case the patient is 37 years old that is in low risk category. If the tumor is larger than 7 cm, the risk of malignancy is about 40%, while for those smaller than 7 cm the risk is about 16% [7]. According to this, our case of 77x82 mm size is borderline.

This soft tissue sarcoma is rare but aggressive and highly resistant malignancy with poor survival. This can be potentiated by power morcellation due to dispersion of tumor [9]. As proven in MITO retrospective group study, power morcellation is the only independent factor associated with survey and increases 3 times the risk of death [10]. To prevent this, morcellation within a bag is much more important as applied in our patient despite her young age. On the other hand, power morcellation decreases survey without illness but the overall survey is not significantly different [10]. In our case the bag was not perforated during the surgery and there was no leakage of sarcoma material into the abdominal cavity. Even morcellation within a bag could not block the dispersion as FDG uptake detected in PET-CT after her second operation. We assumed that one of the main factors associated with dispersion is the myomectomy procedure. Myomectomy may be questioned as a treatment modality because it can be the reason for direct inoculation. Degenerated leiomyomas may be more carefully evaluated and also morcellation may be avoided for especially young patients as in our case.

Although this type of cancer is rarely seen, morcellating it within an isolated bag is important to prevent the worsening of its already adverse consequences. There was no known risk factor involved in this case; however, it is worth noting that any leiomyoma could have a potential risk for cancer. The patient must be informed in detail about this cancer risk even all the risk factors are excluded.

Morcellation within a bag seems to prevent dispersion but even in a benign suggestive case, like ours, dispersion could not be prevented and that again reminds us about myomectomy procedure's role contributing to dispersion before putting the tumor into bag. We assume that only wide dispersion may be prevented with morcellation within a bag as in our case. Using bag may not be preventive due to inoculation during myomectomy via bleeding or seeding with local transmission by touching malign mass.
